# Age- and Sex-Specific Relationships between Household Income, Education, and Diabetes Mellitus in Korean Adults: The Korea National Health and Nutrition Examination Survey, 2008-2010

**DOI:** 10.1371/journal.pone.0117034

**Published:** 2015-01-26

**Authors:** So-Ra Kim, Kyungdo Han, Jin-Young Choi, Jennifer Ersek, Junxiu Liu, Sun-Jin Jo, Kang-Sook Lee, Hyeon Woo Yim, Won-Chul Lee, Yong Gyu Park, Seung-Hwan Lee, Yong-Moon Park

**Affiliations:** 1 Graduate School of Public Health, The Catholic University of Korea, Seoul, Korea; 2 Department of Preventive Medicine, College of Medicine, The Catholic University of Korea, Seoul, Korea; 3 Department of Biostatistics, College of Medicine, The Catholic University of Korea, Seoul, Korea; 4 Catholic Medical Center, The Catholic University of Korea, Seoul, Korea; 5 Department of Epidemiology and Biostatistics, Arnold School of Public Health, The University of South Carolina, Columbia, South Carolina, United States of America; 6 Division of Endocrinology and Metabolism, Department of Internal Medicine, Seoul St.Mary’s Hospital, College of Medicine, The Catholic University of Korea, Seoul, Korea; CUNY, UNITED STATES

## Abstract

**Background:**

To investigate the effects of age and sex on the relationship between socioeconomic status (SES) and the prevalence and control status of diabetes mellitus (DM) in Korean adults.

**Methods:**

Data came from 16,175 adults (6,951 men and 9,227 women) over the age of 30 who participated in the 2008-2010 Korea National Health and Nutrition Examination Survey. SES was measured by household income or education level. The adjusted odds ratios (ORs) and corresponding 95% confidence intervals (95% CI) for the prevalence or control status of diabetes were calculated using multiple logistic regression analyses across household income quartiles and education levels.

**Results:**

The household income-DM and education level-DM relationships were significant in younger age groups for both men and women. The adjusted ORs and 95% CI for diabetes were 1.51 (0.97, 2.34) and 2.28 (1.29, 4.02) for the lowest vs. highest quartiles of household income and education level, respectively, in women younger than 65 years of age (both *P* for linear trend < 0.05 with Bonferroni adjustment). The adjusted OR and 95% CI for diabetes was 2.28 (1.53, 3.39) for the lowest vs. highest quartile of household income in men younger than 65 (*P* for linear trend < 0.05 with Bonferroni adjustment). However, in men and women older than 65, no associations were found between SES and the prevalence of DM. No significant association between SES and the status of glycemic control was detected.

**Conclusions:**

We found age- and sex-specific differences in the relationship of household income and education with the prevalence of DM in Korea. DM preventive care is needed for groups with a low SES, particularly in young or middle-aged populations.

## Introduction

There is a rapidly increasing number of patients with diabetes mellitus (DM) worldwide; in fact, this disease is being described as an ‘epidemic’ [1,2]. The prevalence of DM in South Korea was 1.5% in 1972 and has since increased to 9.1% in 2005 and 9.6% in 2009 [3,4]. According to the annual report from Korea national statistical office, the mortality due to DM reached 207 per million people, which ranked DM as the 5th most common cause of mortality in 2010. These statistics demonstrate that DM is indeed becoming a great concern to national health, particularly with the increased socioeconomic burden in the country.

Environmental factors substantially contribute to the development of DM and are closely related to socioeconomic status (SES). SES is mainly evaluated by educational status, income, and occupation of the subject [5] and has been reported to be linked to dietary habits, exercise frequency, and health behavior [6]. The inverse relationship between SES and alcohol and cigarette use has been well documented, and increased alcohol and cigarette consumption may be related to the higher occurrence of DM [7]. Additionally, it was noted that as SES increases, the likelihood of regular exercise also increases [8]. Furthermore, people with a low SES are more likely to have exposure to toxic substances and are also less likely to have access to appropriate medical care [9]. In this regard, previous studies suggested an inverse relationship between SES and DM [10–12]. A14-year follow-up study in the United States confirmed that DM occurred more frequently in those with a low SES [13].

Recent studies have shown that the association between SES and DM may have cross-country variation [14,15]. The social environment of Korea has changed dramatically along with the rapid industrial development and westernization, which may have greatly influenced the association between SES and DM [16]. However, previous research conducted in the Korean population has only focused on the education level of the subjects [16] or has not considered the possibility of differential associations by age groups [17]. In addition, the association between SES and the control status of DM has not been studied. In the present study, we further explored the relationship between SES and DM by examining both education and household income as indicators of SES by particularly focusing on the effects of age and sex in Korean adults using the representative national data.

## Subjects and Methods

### Study population

Data for this cross-sectional study were collected from the Korea National Health and Nutrition Examination Survey (KNHANES) conducted in 2008–2010. A complex, stratified, multistage probability sampling design based on age, sex, and region was applied in this survey to represent the non-institutionalized civilian Korean population. Details of the surveys performed in KNHANES have been described previously [18,19]. A total of 29,235 participants completed this survey. We excluded individuals younger than 30 years of age because this group is likely to be socioeconomically unstable, especially in their income status. After further exclusion of those with missing data on household income or education level, 16,175 participants remained available for analysis (6,951 men and 9,227 women). The participants were stratified by sex and age group (younger than 65 years or older than 65 years). The reason for categorizing the study subjects by the age of 65 was to consider the possible difference in their working status, growing background and experiencing social transition period. The Institutional Review Board at The Catholic University of Korea approved this study (MC12EASE0054), and written informed consent was obtained from all participants.

### Data collection

Participants were asked about their household income and education level through an interview. They answered questions regarding their age, sex, marital status, history of smoking and drinking, residence and physical activity through a self-administered questionnaire. Place of residence was classified as rural or urban. Marital status was classified as unmarried, married or single (divorce or separated). Occupation was categorized as 1) sales and services; 2) agriculture, forestry, fishery; 3) engineering, assembling, technical work; 4) manual labor; or 5) no job, student or housewife. Smoking status was categorized as current smoker, ex-smoker or never smoked. Data on frequency and amount of alcohol consumed per day were also collected and categorized as non-drinker (≤1 g/day), moderate drinker (1–29.9 g/day) or heavy drinker (≥ 30 g/day). Information on food consumption was obtained via interview using the 24-hour recall method. Total caloric intake and the proportions of energy from carbohydrate, protein and fat were also estimated. Physical activity (regular exercise and walking) was also assessed. Regular exercise was defined as doing moderate exercise (i.e., swimming slowly, tennis, volleyball) for half an hour, 5 or more days per week, or doing intensive exercise (i.e., running, climbing, cycling, swimming fast, football, basketball) for approximately 20 minutes, 3 or more days per week. Participants were classified as walkers if they reported walking for more than 30 minutes at a time at least 5 days per week. The participants’ height, weight and waist circumference in everyday clothing were measured. Height was measured with an accuracy of 0.1 cm using a portable stadiometer (Seca 225; Seca, Hamburg, Germany), and weight was measured to the nearest 0.1 kg using an electronic scale (GL-6000–20; Caskorea, Seoul, Korea). Waist circumference (WC) was measured to the nearest 0.1 cm at the end of expiration; the measurement was made at the midpoint of the lower margin of the ribcage and the iliac crest in the participant’s mid-axillary line using a measuring tape (Seca 200; Seca). Body mass index (BMI) was calculated by dividing weight in kilograms by height in meters squared (kg/m^2^). Blood samples were collected after at least 8 hours of fasting. The specimens were immediately centrifuged, aliquoted, frozen at −70°C and moved to the central laboratory (NeoDIN Medical Institute, Seoul, Korea). The serum levels of glucose, triglycerides and high-density lipoprotein (HDL)-cholesterol were measured enzymatically using an automatic analyzer (Hitachi 7600; Hitachi, Tokyo, Japan). Glycated hemoglobin (HbA1c) levels were analyzed in 1,686 subjects (851 men and 835 women) with DM by high-performance liquid chromatography using HLC-723G7 (Tosoh, Japan).

### Socioeconomic status variables

Household income and education levels were used to assess SES. Monthly income was standardized according to the number of family members (monthly income/√ number of family members) and was divided into 4 quartile groups: lowest, lower middle, higher middle, and highest. Education level was assessed according to the number of years of schooling and classified into four categories: ≤ 6 years (elementary school), 7–9 years (middle school), 10–12 years (high school), and more than 13 years (university).

### Diagnosis of diabetes mellitus

The participants were classified as having DM if they met one of the following conditions: 1) fasting plasma glucose 126 mg/dL or higher, 2) medical diagnosis of DM by a trained medical professional, or 3) treatment with oral hypoglycemic agents or insulin injections. The control status of DM was evaluated by HbA1c levels, with less than 7% being regarded as the optimal level.

### Statistical analysis

All analyses were conducted using SAS version 9.2 (SAS Institute Inc., Cary, NC, USA). The means ± standard error (SE) for the continuous variables or the percentages (SE) for the categorical variables were calculated. A one-way ANOVA or a Rao-Scott chi-square test was used to compare the groups. The SAS survey procedure was applied to reflect the complex sampling design and the sampling weights of KNHANES and to provide nationally representative prevalence estimates. The trend of the relationship between household income and education level with DM was examined using *P* for trend. Multiple logistic regression analyses were used to estimate the prevalence odds ratios (OR), and 95% confidence intervals (CIs) of DM were calculated for each SES category. Several models were applied to evaluate the potential mediation effect of modifiable behaviors such as diet or exercise as well as to consider the effects of known risk factors such as metabolic abnormalities. Thus, model 1 was adjusted for age, place, marital status, smoking, alcohol intake, and education level (across household income) or household income (across education level); model 2 was adjusted further for regular exercise, fat intake, and energy intake; model 3 was further adjusted for BMI, hypertension, high triglycerides (≥ 150 mg/dL), and low HDL-cholesterol (< 40 mg/dL for men and < 50 mg/dL for women). Considering multiple comparisons based on age and gender, a Bonferroni-corrected significance threshold (alpha = 0.01667) was applied. In addition, we conducted stratified analyses to assess effect modification by gender and age on the associations between education and household income and DM. Interaction by sex and age was also evaluated. We assessed three-way interaction between age, sex and either household income or education. We also assessed two-way interaction between age or sex and household income or education.

## Results

Table 1 shows the characteristics of the study population. The mean (± SE) age was 48.6 ± 0.2 years for men and 50.1 ± 0.2 years for women. The prevalence of DM and impaired glucose tolerance were significantly higher in men compared to women. The levels of household income and education were also higher in men compared to women.

**Table 1:** General characteristics of the study participants according to sex.

Variables	Men	Women	*P*
	(n = 6,951)	(n = 9,227)	
Age (years)	48.6 ± 0.2	50.1 ± 0.2	<0.001
Income			<0.001
Lowest	14.6 ± 0.6	17.5 ± 0.6	
Lower middle	24.8 ± 0.7	25.7 ± 0.7	
Higher middle	29.7 ± 0.7	27.8 ± 0.6	
Highest	30.9 ± 0.9	27.0 ± 0.8	
Education			<0.001
≤6 years	15.6 ± 0.6	31.5 ± 0.8	
7–9 years	12.9 ± 0.5	12.0 ± 0.4	
10–12 years	34.9 ± 0.8	34.4 ± 0.7	
≥13 years	36.7 ± 1.0	22.1 ± 0.7	
Place			0.921
Rural	21.4 (1.7)	21.4 (1.6)	
Urban	78.6 (1.7)	78.6 (1.6)	
Marital status			<0.001
Unmarried	8.4 (0.5)	3.2 (0.2)	
Married	86.3 (0.6)	77.3 (0.6)	
Single	5.3 (0.3)	19.5 (0.6)	
Occupation			<0.001
Sales and services	18.9 (0.8)	17.5 (0.6)	
Agriculture/forestry/fishery	13.0 (1.2)	7.4 (0.8)	
Engineering/assembling/technical work	33.5 (1.0)	3.5 (0.3)	
Manual labor	10.4 (0.5)	12.5 (0.4)	
No job/student/housewife	24.3 (0.8)	59.2 (0.8)	
Smoking			<0.001
Never smoked	20.9 (0.6)	91.8 (0.4)	
Ex-smoker	28.7 (0.7)	2.7 (0.2)	
Current	50.4 (0.7)	5.5 (0.3)	
Alcohol intake			<0.001
None	15.2 (0.5)	36.8 (0.7)	
Moderate	65.6 (0.7)	61.7 (0.7)	
Heavy	19.2 (0.6)	1.5 (0.2)	
Energy intake (kcal/day)	2265.5 ± 14.5	1604.5 ± 9.1	<0.001
Fat intake (% of energy)	42.7 ± 0.5	27.8 ± 0.3	
Exercise			<0.001
High intensive exercise			<0.001
Yes	19.2 (0.6)	14.3 (0.5)	
No	80.8 (0.6)	85.7 (0.5)	
Moderate exercise			0.230
Yes	12.9 (0.5)	13.7 (0.5)	
No	87.1 (0.5)	86.3 (0.5)	
Walking exercise			0.136
Yes	43.3 (0.8)	41.8 (0.7)	
No	56.7 (0.8)	58.2 (0.7)	
Body mass index (kg/m^2^)	24.2 ± 0.1	23.6 ± 0.1	<0.001
Waist circumference (cm)	85.4 ± 0.1	79.6 ± 0.2	<0.001
Fasting glucose (mg/dL)	101.9 ± 0.4	97.6 ± 0.3	<0.001
High triglyceride	24.9 (0.6)	11.2 (0.4)	<0.001
Low HDL-cholesterol	22.1 (0.6)	10.5 (0.4)	<0.001
Hypertension	36.1 (0.8)	28.3 (0.6)	<0.001
Diabetes mellitus status			<0.001
Diabetes mellitus	10.9 (0.4)	8.9 (0.4)	
Impaired glucose tolerance	24.7 (0.6)	17 (0.5)	
Normal glucose tolerance	64.4 (0.7)	74 (0.5)	

Values are means ± SE or percentages (SE).

Table 2 shows the distribution of characteristics by household income and education groups for men. A higher household income was associated with a younger age, higher energy and fat intake, higher BMI and WC, but lower prevalence of hypertension and DM. The percentages of urban living, alcohol intake and intensive exercise were higher, while those of smoking and walking were lower, in parallel with increases in household income. This pattern was similar when evaluating education levels; however there were no differences in energy intake according to educational status. The results in men were generally similar to those reported in women (Table 3). However, in contrast to men, BMI and WC were lower in women with higher household incomes and education levels.

**Table 2:** Distribution of characteristics according to socioeconomic status in men.

Variable	Household income					Education level				
	Lowest	Lower middle	Higher middle	Highest	*P*	≤6 years	7–9 years	10–12 years	≥13 years	*P*
	(n = 1,329)	(n = 1,730)	(n = 1,923)	(n = 1,969)		(n = 1,470)	(n = 988)	(n = 2,229)	(n = 2,264)	
Age (years)	59.3 ± 0.5	49.2 ± 0.4	45.5 ± 0.3	46.1 ± 0.3	<0.001	62.6 ± 0.4	55.2 ± 0.4	46.2 ± 0.3	42.7 ± 0.3	<0.001
Place					<0.001					0.001
Rural	32.9 (2.8)	22.8 (2.1)	18.9 (1.9)	16.2 (1.8)		40.1 (2.8)	30.1 (2.6)	20.7 (2.0)	11.0 (1.4)	
Urban	65.1 (2.8)	77.2 (2.1)	81.1 (1.9)	83.8 (1.8)		60.0 (2.8)	69.9 (2.6)	79.3 (2.0)	89.0 (1.4)	
Marital status					<0.001					<0.001
Unmarried	11.0 (1.2)	7.9 (0.9)	7.9 (0.8)	8.0 (1.0)		2.5 (0.5)	4.6 (0.8)	9.6 (0.8)	11.1 (0.9)	
Married	76.6 (1.5)	85.9 (1.2)	89.0 (0.9)	88.6 (1.2)		86.4 (1.2)	87.6 (1.4)	85.7 (1.0)	86.4 (1.0)	
Single	12.4 (1.2)	6.2 (0.7)	3.1 (0.5)	3.4 (0.5)		11.2 (1.1)	7.8 (1.1)	4.7 (0.5)	2.5 (0.4)	
Occupation					<0.001					<0.001
Sales and services	6.2 (0.9)	16.2 (1.1)	24.5 (1.4)	26.1 (1.8)		6.1 (0.9)	11.7 (1.3)	20.8 (1.2)	35.2 (1.8)	
Agriculture/forestry/fishery	18.7 (1.9)	13.6 (1.5)	9.5 (1.3)	11.6 (1.8)		26.0 (2.3)	18.0 (2.0)	7.7 (1.1)	4.8 (1.0)	
Engineering/assembling/technical work	13.5 (1.1)	35.5 (1.5)	41.0 (1.7)	38.7 (2.1)		17.6 (1.5)	34.8 (2.0)	45.3 (1.6)	26.4 (1.8)	
Manual labor	13.1 (1.2)	10.3 (1.0)	10.6 (1.0)	8.0 (1.0)		15.1 (1.3)	12.2 (1.4)	9.0 (0.8)	6.5 (1.0)	
No job/student/housewife	48.5 (1.9)	24.4 (1.3)	14.4 (1.1)	15.6 (1.4)		35.2 (1.7)	23.4 (1.7)	17.2 (1.0)	27.1 (1.8)	
Smoking					<0.001					<0.001
Never smoked	20.2 (1.6)	17.2 (1.1)	20.0 (1.1)	24.7 (1.1)		17.12 (1.3)	17.9 (1.5)	17.6 (0.9)	26.6 (1.1)	
Ex-smoker	30.4 (1.5)	27.4 (1.2)	28.0 (1.2)	29.5 (1.2)		35.6 (1.7)	30.7 (1.9)	27.5 (1.1)	26.2 (1.0)	
Current	49.5 (1.9)	54.9 (1.52)	52.0 (1.3)	45.8 (1.4)		47.3 (1.7)	51.4 (2.1)	54.9 (1.3)	47.1 (1.2)	
Alcohol intake					<0.001					<0.001
None	26.6 (1.3)	17.9 (1.1)	12.5 (0.9)	10.1 (0.8)		26.5 (1.5)	17.6 (1.4)	12.0 (0.8)	12.5 (0.8)	
Moderate	55.1 (1.6)	62.3 (1.4)	68.6 (1.3)	70.5 (1.2)		51.3 (1.6)	60.6 (1.9)	67.5 (1.1)	71.7 (1.1)	
Heavy	18.3 (1.4)	19.8 (1.1)	18.9 (1.0)	19.5 (1.0)		22.2 (1.4)	21.9 (1.6)	20.5 (1.0)	15.8 (0.9)	
Energy intake (kcal/day)	2102.4 ± 34.5	2200.6 ± 27.6	2292.5 ± 26.3	2302.6 ± 26.7	<0.001	2191.3 ± 34.8	2273.1 ± 26.0	2210.2 ± 31.1	2241.0 ± 24.7	0.279
Fat intake (% of energy)	36.5 ± 1.1	39.6 ± 0.9	43.2 ± 1.0	44.5 ± 1.0	<0.001	34.8 ± 0.9	43.5 ± 1.0	38.2 ± 1.2	45.7 ± 1.0	<0.001
Exercise										
High intensive exercise					<0.001					0.002
Yes	12.7 (1.2)	19.2 (1.2)	20.0 (1.0)	21.7 (1.1)		15.5 (1.21)	20.8 (1.6)	21.8 (1.0)	17.8 (0.9)	
No	87.3 (1.2)	80.8 (1.2)	80.0 (1.0)	78.3 (1.1)		84.5 (1.2)	79.2 (1.6)	78.2 (0.9)	82.2 (0.9)	
Moderate exercise					0.729					<0.001
Yes	11.8 (1.2)	13.5 (1.0)	12.8 (0.9)	13.1 (0.8)		14.8 (1.2)	14.0 (1.3)	14.6 (0.9)	10.1 (0.7)	
No	88.2 (1.2)	86.6 (1.0)	87.2 (0.9)	86.9 (0.8)		85.2 (1.2)	86.0 (1.3)	85.4 (0.9)	89.9 (0.7)	
Walking exercise					0.004					<0.001
Yes	48.8 (1.8)	44.1 (1.5)	41.6 (1.3)	41.5 (1.3)		51.1 (1.6)	41.8 (1.9)	44.6 (1.2)	39.2 (1.2)	
No	51.2 (1.8)	55.9 (1.5)	58.4 (1.3)	58.5 (1.3)		48.9 (1.6)	58.2 (1.9)	55.4 (1.2)	60.8 (1.2)	
Body mass index (kg/m^2^)	23.6 ± 0.1	24.0 ± 0.1	24.1 ± 0.1	24.3 ± 0.1	<0.001	23.5 ± 0.1	24.1 ± 0.1	24.0 ± 0.1	24.4 ± 0.1	<0.001
Waist circumference (cm)	84.0 ± 0.3	85.2 ± 0.3	85.0 ± 0.2	85.8 ± 0.2	<0.001	83.8 ± 0.3	85.0 ± 0.2	85.3 ± 0.3	85.7 ± 0.2	<0.001
Fasting glucose (mg/dL)	102.2 ± 1.1	101.6 ± 1.0	102.6 ± 0.8	101.2 ± 0.5	0.411	99.0 ± 0.8	102.8 ± 0.7	103.6 ± 1.0	101.9 ± 0.7	0.001
High triglyceride	23.8 (1.4)	25.6 (1.4)	25.1 (1.2)	24.8 (1.1)	0.8406	22.8 (1.5)	28.7 (1.7)	25.9 (1.1)	23.6 (1.0)	0.0233
Low HDL-cholesterol	24.8 (1.4)	22.2 (1.3)	22.1 (1.2)	20.7 (1.0)	0.1802	24.4 (1.4)	26.8 (1.7)	20.8 (1.0)	20.7 (1.0)	0.0014
Hypertension	47.2 (1.8)	35.6 (1.4)	33.5 (1.3)	33.9 (1.3)	<.0001	48.3 (1.8)	43.1 (1.9)	35.4 (1.2)	29.3 (1.2)	<.0001
Diabetes mellitus	20.0 (1.4)	11.2 (0.9)	8.9 (0.8)	8.4 (0.7)	<0.001	17.1 (1.1)	16.3 (1.3)	9.9 (0.7)	7.5 (0.6)	<0.001

Values are means ± SE or percentages (SE).

Obtained by ANOVA for continuous variables and by chi-square test for categorical variables.

**Table 3:** Distribution of characteristics according to socioeconomic status in women.

Household income						Education level				
Variable	Lowest	Lower middle	Higher middle	Highest	*P*	≤6 years	7–9 years	10–12 years	≥13 years	*P*
	(n = 2,136)	(n = 2,290)	(n = 2,426)	(n = 2,375)		(n = 3,469)	(n = 1,059)	(n = 2,838)	(n = 1,861)	
Age (year)	61.6 ± 0.4	49.8 ± 0.3	46.1 ± 0.3	46.5 ± 0.3	<0.001	64.0 ± 0.3	52.1 ± 0.3	43.5 ± 0.2	39.7 ± 0.3	<0.001
Place					<0.001					<0.001
Rural	35.2 (2.6)	21.6 (2.0)	18.1 (1.8)	14.7 (1.6)		35.0 (2.4)	24.8 (2.6)	15.0 (1.6)	10.2 (1.4)	
Urban	64.8 (2.6)	78.4 (2.0)	81.9 (1.8)	85.3 (1.6)		65.0 (2.4)	75.2 (2.6)	85.0 (1.6)	89.8 (1.4)	
Marital status					<0.001					<0.001
Unmarried	2.6 (0.5)	2.8 (0.4)	3.9 (0.5)	3.1 (0.5)		0.7 (0.2)	1.0 (0.4)	2.7 (0.4)	8.5 (0.8)	
Married	54.0 (0.34)	76.9 (1.1)	83.9 (0.9)	87.8 (0.9)		58.9 (1.1)	81.4 (1.4)	86.4 (0.9)	87.3 (1.0)	
Single	43.3 (1.3)	20.4 (1.0)	12.2 (0.8)	9.17 (0.7)		40.4 (1.1)	17.6 (1.4)	10.9 (0.8)	4.3 (0.5)	
Occupation					<0.001					<0.001
Sales and services	8.9 (0.8)	19.0 (1.2)	21.8 (1.1)	18.9 (1.2)		9.0 (0.7)	23.2 (1.6)	26.0 (1.1)	13.7 (1.3)	
Agriculture/forestry/fishery	12.3 (1.2)	7.3 (0.9)	5.5 (0.8)	5.3 (0.9)		14.8 (1.4)	8.3 (1.5)	2.5 (0.5)	0.6 (0.3)	
Engineering/assembling/technical work	2.1 (0.4)	4.0 (0.5)	4.4 (0.5)	3.0 (0.5)		3.0 (0.4)	7.1 (1.0)	3.7 (0.4)	1.0 (0.3)	
Manual labor	14.1 (0.9)	15.9 (1.0)	11.8 (0.8)	7.8 (0.8)		14.9 (0.7)	19.0 (15.1)	11.8 (0.8)	2.9 (0.6)	
No job/student/housewife	62.6 (1.4)	53.9 (1.4)	56.5 (1.4)	65.0 (1.6)		58.3 (1.3)	42.4 (27.7)	56.1 (1.3)	81.8 (1.5)	
Smoking					<0.001					<0.001
Never smoked	89.6 (0.8)	90.3 (0.8)	92.6 (0.6)	94.0 (0.6)		91.0 (0.7)	92.0 (1.2)	90.2 (0.7)	95.5 (0.6)	
Ex-smoker	3.6 (0.5)	2.3 (0.4)	3.1 (0.4)	2.1 (0.3)		3.3 (0.3)	1.3 (0.4)	2.9 (0.3)	2.2 (0.4)	
Current	6.80 (0.7)	7.4 (0.7)	4.3 (0.5)	4.0 (0.5)		5.7 (0.6)	6.7 (1.10)	6.9 (0.6)	2.3 (0.4)	
Alcohol intake					<0.001					<0.001
None	51.5 (1.4)	37.0 (1.3)	32.7 (1.1)	30.2 (1.2)		52.7 (1.2)	31.9 (1.5)	27.8 (1.1)	30.7 (1.3)	
Moderate	46.7 (1.4)	61.8 (1.3)	65.1 (1.8)	69.1 (1.5)		46.3 (1.2)	66.1 (1.6)	7.0 (1.1)	68.6 (1.3)	
Heavy	1.9 (0.4)	1.2 (0.3)	2.2 (0.4)	0.7 (0.2)		1.1 (0.2)	2.0 (0.6)	2.2 (0.3)	0.6 (0.2)	
Energy intake (kcal/day)	1523.5 ± 18.2	1608.7 ± 16.4	1640.9 ± 15.7	1684.6 ± 17.4	<0.001	1531.0 ± 19.1	1643.9 ± 15.3	1651.0 ± 22.9	1720.3 ± 20.7	<0.001
Fat intake (% of energy)	24.9 ± 0.5	27.9 ± 0.6	29.1 ± 0.5	31.4 ± 0.5	<0.001	22.7 ± 0.51	31.2 ± 0.56	27.31 ± 0.76	35.49 ± 0.74	<0.001
Exercise										
High intensive exercise					<0.001					<0.001
Yes	10.8 (0.8)	13.4 (1.0)	15.5 (0.9)	16.9 (1.0)		10.5 (0.7)	17.8 (1.3)	16.7 (0.9)	14.1 (0.9)	
No	89.9 (0.8)	86.6 (1.0)	84.5 (0.9)	83.1 (1.0)		89.5 (0.9)	82.2 (1.3)	83.3 (0.9)	85.9 (0.9)	
Moderate exercise					0.147					<0.001
Yes	11.7 (0.8)	14.2 (1.0)	14.6 (0.9)	13.6 (0.9)		14.9 (0.8)	17.7 (1.5)	13.3 (0.8)	10.4 (0.9)	
No	88.3 (0.8)	85.8 (1.0)	85.4 (0.9)	86.4 (0.9)		85.1 (0.8)	82.3 (1.5)	86.7 (0.8)	89.6 (0.9)	
Walking exercise					0.080					<0.001
Yes	41.1 (1.4)	44.5 (1.2)	41.1 (1.2)	40.5 (1.3)		42.8 (1.2)	46.4 (1.8)	42.7 (1.2)	36.6 (1.4)	
No	58.9 (1.4)	55.5 (1.2)	58.9 (1.2)	59.5 (1.3)		57.2 (1.2)	53.6 (1.8)	57.3 (1.2)	63.4 (1.4)	
Body mass index (kg/m^2^)	23.9 ± 0.1	24.0 ± 0.1	23.6 ± 0.1	23.3 ± 0.1	<0.001	24.3 ± 0.1	23.4 ± 0.1	24.3 ± 0.1	22.6 ± 0.1	<0.001
Waist circumference (cm)	80.6 ± 0.3	80.5 ± 0.3	79.7 ± 0.2	78.5 ± 0.2	<0.001	82.3 ± 0.3	78.6 ± 0.2	80.8 ± 0.3	76.3 ± 0.3	<0.001
Fasting glucose (mg/dL)	97.9 ± 0.8	99.4 ± 0.6	97.1 ± 0.5	96.2 ± 0.4	<0.001	99.8 ± 0.7	97.3 ± 0.5	97.6 ± 0.7	94.1 ± 0.5	<0.001
High triglyceride	17.5 (1.1)	12.5 (0.8)	9.4 (0.7)	7.3 (0.6)	<.0001	18.7 (0.8)	10.7 (1.1)	8.5 (0.6)	4.9 (0.6)	<.0001
Low HDL-cholesterol	15.1 (0.9)	11.1 (0.8)	9.6 (0.7)	7.7 (0.6)	<.0001	16.8 (0.8)	10.1 (1.0)	7.4 (0.5)	6.7 (0.7)	<.0001
Hypertension	49.4 (1.3)	26.9 (1.1)	22.4 (1.1)	20.4 (1.1)	<.0001	54.2 (1.1)	32.0 (1.7)	16.5 (0.8)	7.6 (0.8)	<.0001
Diabetes mellitus	15.9 (1.0)	10.3 (0.7)	6.0 (0.7)	5.7 (0.6)	<0.001	18.2 (0.9)	7.5 (0.9)	5.4 (0.5)	2.2 (0.5)	<0.001

Values are means ± SE or percentages (SE).

Obtained by ANOVA for continuous variables and by chi-square test for categorical variables.

**Fig 1:**
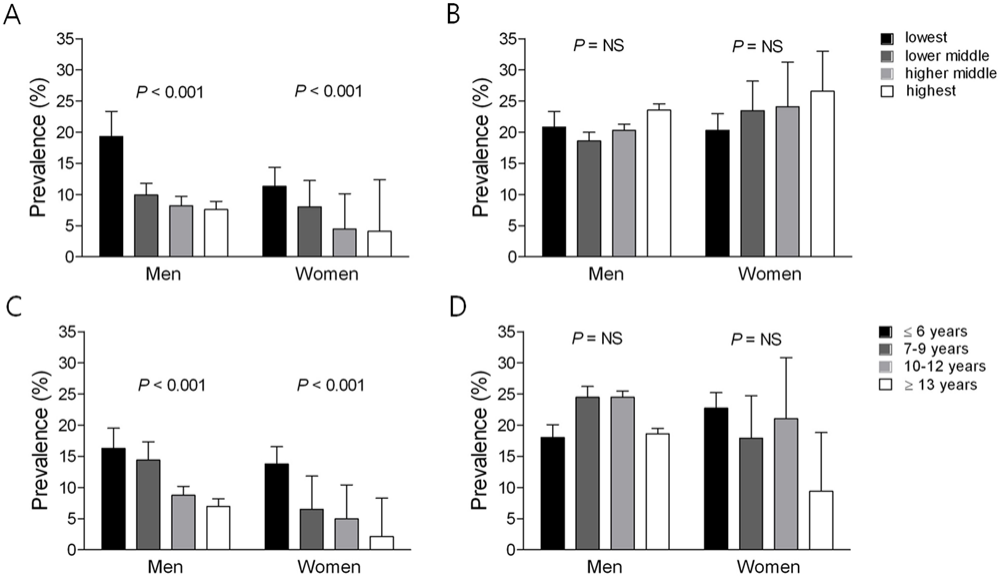
The prevalence of diabetes mellitus according to the level of household income in participants less than (A) or greater than (B) 65 years old and to the level of education in participants less than (C) or greater than (D) 65 years old. NS, non-specific.

None of the three-way interactions we evaluated were significant. However, there was significant interaction by sex on the relationship between education and DM (*P* < 0.001) as well as between household income and DM (*P* = 0.009). There was also significant interaction by age group on the relationship between education and DM (*P* = 0.001) and on the relationship between household income and DM (*P* < 0.001). This supported the rationale for stratified analyses by gender and age group (S1 Fig.). Next, we investigated the prevalence of DM in age- and sex-specific groups according to household income and education. In men less than 65 years old, the prevalence of DM in the lowest, lower middle, higher middle, and highest household income groups was 19.3%, 10.0%, 8.2%, and 7.6% (*P* for trend < 0.001), respectively. The corresponding percentages of DM in women less than 65 years of age were 11.3%, 8.0%, 4.5%, and 4.1% (*P* for trend < 0.001), respectively (Fig. 1A). However, there were no differences in the prevalences of DM in either men or women over 65 years old according to the household income status (Fig. 1B). In men less than 65 years old, the prevalence of DM was lower with higher education levels (16.3% with less than 6 years of education, 14.4% with 7–9 years of education, 8.8% with 9–12 years of education and 7.0% with over 13 years of education; *P* for trend < 0.001). The corresponding percentages of DM in women less than 65 years of age were 13.8%, 6.5%, 5.0%, and 2.1% (*P* for trend < 0.001), respectively (Fig. 1C). However, this tendency was not observed in men or women over 65 years old (Fig. 1D).

The OR for DM according to household income and education was analyzed by age- and sex-specific groups (Table 4). When compared model 1 without potential mediators and model 2 with potential mediators, no discernible differences were observed in the ORs. After additional adjustment for BMI, hypertension, high triglycerides, and low HDL-cholesterol, in men and women less than 65 years of age, the adjusted ORs (95% CI) of DM for the lowest vs. highest quartile of household income were 2.28 (1.53, 3.39) and 1.51 (0.97, 2.34), respectively. This demonstrated the trend that a lower household income level was associated with a higher prevalence of DM (both *P* for linear trend < 0.05 with Bonferroni adjustment). In addition, in women less than 65 years of age, the adjusted OR (95% CI) of DM for the lowest vs. highest level of education was 2.28 (1.29, 4.02), suggesting that a lower education level was associated with a higher prevalence of DM (*P* for linear trend < 0.05 with Bonferroni adjustment). In men less than 65 years of age, the unadjusted analysis showed significant association between education level and the prevalence of DM. However, this relationship disappeared after adjusting for multiple variables including household income. In men and women over 65 years of age, no associations were found between SES and the prevalence of DM.

**Table 4:** Age- and sex-specific odds ratios (95% CIs) for diabetes mellitus according to socioeconomic status.

Household income						Education level				
	Highest	Higher middle	Lower middle	Lowest	*P* for trend	≥13 years	10–12 years	7–9 years	≤6 years	*P* for trend
Men										
Total										
*n*	1,969	1,923	1,730	1,329		2,264	2,229	988	1,470	
Age-adjusted	1 (ref)	1.07 (0.83,1.38)	1.39 (1.09,1.77)	2.74 (2.16,3.48)	<0.001	1 (ref)	1.35 (1.08,1.68)	2.41 (1.86,3.12)	2.53 (1.99,3.23)	<0.001
Model 1	1 (ref)	1.11 (0.84,1.48)	1.24 (0.93,1.65)	1.53 (1.10,2.12)	0.084	1 (ref)	1.02 (0.79,1.31)	1.13 (0.81,1.58)	0.86 (0.59,1.24)	0.292
Model 2	1 (ref)	1.13 (0.83,1.53)	1.21 (0.83,1.53)	1.46 (1.02,2.09)	0.045	1 (ref)	1.23 (0.85,1.79)	1.02 (0.76,1.37)	0.94 (1.62,1.43)	0.938
Model 3	1 (ref)	1.07 (0.81,1.42)	1.11 (0.83,1.49)	1.47 (1.05,2.06)	0.042	1 (ref)	1.11 (0.85,1.45)	1.36 (0.97,1.92)	1.00 (0.68,1.46)	0.8085
<65 years										
*n*	1,799	1,704	1,272	529		2,.017	1,922	694	617	
Age-adjusted	1 (ref)	1.08 (0.82,1.43)	1.34 (1.02,1.78)	2.92 (2.15,3.92)	<0.001	1 (ref)	1.27 (1.00,1.62)	2.25 (1.65,3.06)	2.59 (1.91,3.53)	<0.001
Model 1	1 (ref)	1.16 (0.84,1.61)	1.38 (1.00,1.90)	2.28 (1.55,3.35)	<0.001	1 (ref)	0.83 (0.63,1.09)	0.73 (0.48,1.10)	0.69 (0.45,1.04)	0.310
Model 2	1 (ref)	1.15 (0.81,1.64)	1.31 (0.91,1.89)	2.18 (1.42,3.33)	0.001	1 (ref)	0.79 (0.49,1.26)	0.94 (0.67,1.31)	0.84 (0.60,1.16)	0.303
Model 3	1 (ref)	1.19 (0.86,1.64)	1.26 (0.89,1.78)	2.28 (1.53,3.39)	0.001	1 (ref)	0.95 (0.69,1.30)	0.99 (0.65,1.52)	0.91 (0.58,1.43)	0.730
≥65 years										
*n*	170	219	458	800		193	307	294	853	
Age-adjusted	1 (ref)	0.82 (0.43,1.48)	0.75 (0.43,1.30)	0.87 (0.53,1.43)	0.765	1 (ref)	1.44 (0.87,2.40)	1.41 (0.88,2.27)	0.97 (0.62,1.51)	0.059
Model 1	1 (ref)	1.31 (0.67,2.55)	1.12 (0.59,2.10)	1.33 (0.74,2.38)	0.694	1 (ref)	2.03 (1.12,3.71)	1.99 (1.12,3.52)	1.59 (0.92,2.76)	0.078
Model 2	1 (ref)	1.45 (0.72,2.92)	1.16 (0.60,2.26)	1.33 (0.72,2.47)	0.528	1 (ref)	1.92 (1.02,3.61)	2.03 (1.11,3.72)	1.63 (0.90,2.95)	0.550
Model 3	1 (ref)	0.71 (0.39,1.30)	0.68 (0.39,1.18)	0.83 (0.51,1.37)	0.940	1 (ref)	1.47 (0.83,2.59)	1.51 (0.89,2.57)	1.14 (0.67,1.92)	0.784
Women										
Total										
*n*	2,375	2,426	2,290	2,136		1,861	2,838	1,059	3,469	
Age-adjusted	1 (ref)	1.06 (0.78,1.45)	1.91 (1.49,2.44)	3.08 (2.39,3.98)	<0.001	1 (ref)	2.54 (1.62,3.99)	3.65 (2.25,5.92)	9.87 (6.46,15.09)	<0.001
Model 1	1 (ref)	0.98 (0.71,1.35)	1.32 (1.03,1.69)	1.16 (0.87,1.55)	0.069	1 (ref)	2.09 (1.32,3.30)	2.11 (1.28,3.50)	3.33 (2.03,5.47)	<0.001
Model 2	1 (ref)	1.03 (0.73,1.43)	1.39 (1.06,1.81)	1.21 (0.90,1.64)	0.085	1 (ref)	2.32 (1.35,3.97)	2.22 (1.35,3.66)	3.62 (2.13, 6.17)	<0.001
Model 3	1 (ref)	1.02 (0.72,1.44)	1.36 (1.04,1.78)	1.12 (0.82,1.53)	0.209	1 (ref)	2.08 (1.25,3.48)	1.73 (0.99,3.03)	2.89 (1.67,4.97)	<0.001
<65 years										
*n*	2,151	2,160	1,812	875		1,827	2,724	923	1,524	
Age-adjusted	1 (ref)	1.10 (0.77,,1.56)	2.07 (1.53,2.80)	3.00 (2.10,4.31)	<0.001	1 (ref)	2.43 (1.56,3.75)	3.23 (1.98,5.30)	7.38 (4.81,11.33)	<0.001
Model 1	1 (ref)	1.03 (0.72,1.49)	1.58 (1.14,2.17)	1.67 (1.11,2.52)	0.003	1 (ref)	1.76 (1.12,2.77)	1.47 (0.86,2.53)	2.18 (1.31,3.64)	0.009
Model 2	1 (ref)	1.11 (0.76,1.62)	1.67 (1.19,2.35)	1.70 (1.11,2.61)	0.001	1 (ref)	1.89 (1.16,3.08)	1.58 (0.89,2.79)	2.39 (1.39, 4.10)	0.006
Model 3	1 (ref)	1.07 (0.72,1.59)	1.60 (1.13,2.27)	1.51 (0.97,2.34)	0.006	1 (ref)	1.86 (1.12,3.09)	1.32 (0.73,2.39)	2.28 (1.29,4.02)	0.0164
≥65 years										
*n*	224	266	478	1,261		34	114	136	1,945	
Age-adjusted	1 (ref)	0.89 (0.53,1.43)	0.86 (0.57,1.31)	0.70 (0.20,0.99)	0.020	1 (ref)	2.58 (0.57,11.68)	2.13 (0.50,9.02)	2.81 (0.71, 11.19)	0.333
Model 1	1 (ref)	0.84 (0.49,1.45)	0.81 (0.53,1.26)	0.69 (0.47,1.00)	0.190	1 (ref)	2.99 (0.64,14.06)	2.66 (0.61,11.59)	4.02 (0.97,16.67)	0.102
Model 2	1 (ref)	0.82 (0.48,1.41)	0.81 (0.52,1.26)	0.70 (0.47,1.04)	0.081	1 (ref)	2.68 (0.56,12.78)	2.43 (0.54,10.86)	3.41 (0.80,14.46)	0.080
Model 3	1 (ref)	0.86 (0.50,1.47)	0.85 (0.55,1.32)	0.70 (0.48,1.04)	0.078	1 (ref)	2.79 (0.63,12.46)	2.21 (0.52,9.41)	3.20 (0.80,12.86)	0.138

Adjusted ORs (model 1) for diabetes were determined after adjusting for age, place, marital status, smoking, alcohol intake, and education level (across household income) or household income (across education level).

Adjusted ORs (model 2) for diabetes were determined by additionally adjusting for regular exercise, fat intake, and energy intake.

Adjusted ORs (model 3) for diabetes were determined by additionally adjusting for body mass index, hypertension, high triglycerides, and low HDL-cholesterol.

OR, odds ratio; CI, confidence interval.

The control rate of DM, defined as an HbA1c level of less than 7%, was assessed in subjects with DM. In both men and women, and in both age groups, no significant differences in control rate were noted according to household income and education subgroups (S1 and S2 Tables, S2 and S3 Figs.).

## Discussion

The present study showed a significant relationship between household income and DM, as well as a significant relationship between education level and DM, in younger age groups for men and women. In men younger than 65, household income level was inversely associated with the prevalence of DM. In women younger than 65, household income and education level were both inversely associated with the prevalence of DM. However, these associations were not observed in the older age group.

SES has different influences according to sex and age, and studies examining the relationship between SES and DM have reported that the relationship varied depending on sex, race and the degree of development of societies and countries [11,20,21]. A study regarding the factors that affect the difference between the sexes, explained that this occurs because the impacts of household income and education are different in men and women [22,23].

In this study, household income levels in young or middle aged men and women younger than 65 were found to be correlated with the prevalence of DM. A low household income is known to be associated with a variety of low health status indicators, such as low birth weight, early childhood mortality, and adult mortality [24]. It also has been reported that people with lower levels of income have limited resources; thus, they would not have a wide range of choices for food or the economic ability to conduct activities that are helpful for health; they have also been reported to have a high degree of psychosocial stress [25]. Because the low income group may not be able to afford health-related activities while those with higher incomes are able to afford these activities, people with a higher income level are reported to have a higher ability to control their health [26,27].

Similarly, education level in young or middle aged women less than 65 years old was associated with DM. This result was similar to results in other studies reporting that a low education level is related to DM in women but not in men [28–30]. Women with a higher SES have been shown to eat adequate foods [31,32] and manage their weight through regular exercise and by checking their health status periodically. Education affects the acquisition and comprehension of health knowledge, and women with higher levels of education have easier access to information and resources that are helpful for health improvement; additionally, women in general have a higher level of interest in health issues than men. It has also been reported that women make a greater effort to conduct healthy living habits when they were given SES indulgence compared with men [33,34], and women with a lower SES have more psychosocial stress than men [35,36]. This coincides with a study stating that a low SES in women would cause more negative impacts on health than a low SES in men [37]. It has also been reported that cardiovascular disease and metabolic syndrome, which are related to DM, were also associated with a low SES in women [38,39].

When stratifying by age, neither household income nor education level was associated with DM in elderly people over 65 years of age, which contrasts with the results observed in the younger age group. Several possible explanations could be considered regarding this phenomenon. Because the development of DM is highly dependent on aging, people aged 65 years and older may be greatly influenced by their physical status rather than by their health habits or external factors. It is also believed that the difficulty of conducting an accurate measurement of income level due to the changing status of working and levels of income might influence the results in the older group [40]. Previous studies have reported that younger age groups may learn healthy living habits more easily than older groups [41]. In addition, young adults and middle-aged people comprise the age group that is primarily responsible for earning a living; this age group also deals with a lot of stress. In fact, it has been reported that younger people with lower levels of income may overlook their health [42]. Thus, the influence of SES might be greater in younger populations compared to older ones.

The findings in this study should also be interpreted while considering the rapid changes in the social environment of Korea [16]. Korea’s elderly population was born during underdeveloped period, and most of those people have not received formal education and have spent most of their lifetime during periods in which the national SES was low. The younger age group worked actively in the period of rapid industrial development during the late 20th century. Our data show a large difference in the distribution of household income or education level between the two age groups. Most of the individuals in the older age group had lower levels of household income and education. Therefore, it was expected that the younger age group, which had experienced more dynamic changes in economic development and received more education, was more likely to be affected by SES.

Unexpectedly, we did not observe any associations between the status of DM control and household income or education, which contrasts with the results of previous studies [43,44]. Although we do not have a clear answer at this time, the wide coverage from national health insurance and the nationwide management program for DM provided by public health centers in Korea might have lowered barriers to treatment and may have given equal opportunity for the treatment of DM, independent of a person’s SES. However, further studies in conjunction with various factors affecting glycemic control (e.g., treatment modality, adherence to treatment, medical cost) need to be performed.

This study has several limitations. First, it is a cross-sectional study, which makes it difficult to address the temporal sequence of DM and income status or education; prospective studies are needed to better understand the relationship between SES and DM. Second, using the data generated by KNHANES, we were not able to identify the type of DM. Therefore, we confined the subjects to those aged 30 and over in an effort to reduce the possibility of including type 1 DM. Third, this study utilized the level of household income and education as the indices to represent SES; however, it is insufficient to evaluate SES with only those indices. Fourth, the small sample size in the elderly women with higher education made a relative standard error greater than 30%, which could result in unreliable estimates. We also did not include an index considering organizations to which individuals belong and features of local communities (an indicator of social support), which represents another limitation.

## Conclusions

This study observed a relationship of household income and education with DM in Korea using nationally representative data in adults between 30 and 65 years of age. These findings highlight the inequality of health according to SES in the younger population. With the increasing prevalence of DM and the fact that SES is one of the most important factors determining one’s lifestyle, further study examining the effects of SES on DM is essential. Furthermore, preventive care is needed for groups with low SES, particularly in the young or middle-aged populations.

## Supporting Information

S1 Table Age- and sex-specific odds ratios (95% CIs) for the higher HbA1c levels (≥ 7%) according to household income levels. (DOCX)

S2 Table Age- and sex-specific odds ratios (95% CIs) for the higher HbA1c levels (≥ 7%) according to education levels. (DOCX)

S1 Fig Effect modification by age group (A) and gender (B) on the relationship between household income, education and DM. (TIF)

S2 Fig The control rate of DM (HbA1c < 7%) according to the level of household income in participants less than (A) or greater than (B) 65 years old. NS, non-specific.
(TIF)

S3 Fig The control rate of DM (HbA1c < 7%) according to the level of education in participants less than (A) or greater than (B) 65 years old. NS, non-specific.
(TIF)
