# Supplementary material for: Age- and Sex-Specific Relationships between Household Income, Education, and Diabetes Mellitus in Korean Adults: The Korea National Health and Nutrition Examination Survey, 2008-2010
Source: PLoS One. 2015 Jan 26;10(1):e0117034. doi: 10.1371/journal.pone.0117034 (PMC4306546; doi:10.1371/journal.pone.0117034)
Supplement: S1 Table — (DOCX) [file pone.0117034.s001.docx]

| **Table S1**. Age- and sex-specific odds ratios (95% CIs) for the higher HbA1c levels (≥ 7%) according to household income levels | | | | | |
| --- | --- | --- | --- | --- | --- |
|  | Highest | Higher middle | Lower middle | Lowest | *P* for trend |
| Men |  |  |  |  |  |
| Total |  |  |  |  |  |
| *n* | 181 | 195 | 209 | 266 |  |
| Age-adjusted | 1 (ref) | 1.37 (0.83,2.24) | 1.07 (0.66,1.73) | 1.05 (0.65,1.70) | 0.9311 |
| Model1 | 1 (ref) | 1.43 (0.86,2.37) | 1.10 (0.68,1.79) | 1.11 (0.67,1.85) | 0.9271 |
| Model2 | 1 (ref) | 1.25 (0.70,2.22) | 0.94 (0.54,1.64) | 0.97 (0.53,1.78) | 0.6863 |
| Model3 | 1 (ref) | 1.33 (0.74,2.39) | 0.94 (0.53,1.67) | 1.01 (0.55,1.87) | 0.7321 |
| <65 years |  |  |  |  |  |
| *N* | 146 | 147 | 124 | 103 |  |
| Age-adjusted | 1 (ref) | 1 (ref) | 1 (ref) | 0.98 (0.58,1.66) | 0.9360 |
| Model1 | 1 (ref) | 1 (ref) | 1 (ref) | 0.97 (0.54,1.77) | 0.9312 |
| Model2 | 1 (ref) | 1 (ref) | 1 (ref) | 0.98 (0.54,1.80) | 0.9570 |
| Model3 | 1 (ref) | 1 (ref) | 1 (ref) | 0.97 (0.54,1.76) | 0.9248 |
| ≥65 years |  |  |  |  |  |
| *n* | 35 | 48 | 85 | 163 |  |
| Age-adjusted | 1 (ref) | 1 (ref) | 1 (ref) | 1.32 (0.78,2.19) | 0.2803 |
| Model1 | 1 (ref) | 1 (ref) | 1 (ref) | 1.08 (0.58,2.00) | 0.8076 |
| Model2 | 1 (ref) | 1 (ref) | 1 (ref) | 1.08 (0.58,2.02) | 0.8015 |
| Model3 | 1 (ref) | 1 (ref) | 1 (ref) | 1.02 (0.55,1.89) | 0.9508 |
| Women |  |  |  |  |  |
| Total |  |  |  |  |  |
| *n* | 123 | 138 | 247 | 327 |  |
| Age-adjusted | 1 (ref) | 1.24 (0.68,2.27) | 1.11 (0.65,1.91) | 1.06 (0.63,1.79) | 0.3813 |
| Model1 | 1 (ref) | 1.18 (0.64,2.18) | 1.01 (0.58,1.77) | 1.01 (0.59,1.71) | 0.8757 |
| Model2 | 1 (ref) | 1.31 (0.69,2.48) | 1.11 (0.62,1.98) | 1.10 (0.62,1.93) | 0.9561 |
| Model3 | 1 (ref) | 1.31 (0.68,2.52) | 1.13 (0.62,2.04) | 1.13 (0.64,1.99) | 0.8692 |
| <65 years | | | | | |
| *n* | 72 | 90 | 141 | 100 |  |
| Age-adjusted | 1 (ref) | 1 (ref) | 1 (ref) | 0.92 (0.52,1.64) | 0.7858 |
| Model1 | 1 (ref) | 1 (ref) | 1 (ref) | 1.01 (0.55,1.88) | 0.9643 |
| Model2 | 1 (ref) | 1 (ref) | 1 (ref) | 0.97 (0.51,1.82) | 0.9155 |
| Model3 | 1 (ref) | 1 (ref) | 1 (ref) | 1.03 (0.54,1.95) | 0.9351 |
| ≥65 years | |  |  |  |  |
| *n* | 51 | 48 | 106 | 227 |  |
| Age-adjusted | 1 (ref) | 1 (ref) | 1 (ref) | 1.10 (0.70,1.75) | 0.6757 |
| Model1 | 1 (ref) | 1 (ref) | 1 (ref) | 1.07 (0.66,1.73) | 0.1878 |
| Model2 | 1 (ref) | 1 (ref) | 1 (ref) | 1.10 (0.68,1.78) | 0.7104 |
| Model3 | 1 (ref) | 1 (ref) | 1 (ref) | 1.05 (0.65,1.71) | 0.8433 |

Adjusted ORs (model 1) were determined after adjusting for age, place, marital status, smoking, alcohol intake, and education level.

Adjusted ORs (model 2) were determined by additionally adjusting for regular exercise, fat intake, and energy intake.

Adjusted ORs (model 3) were determined by additionally adjusting for body mass index, hypertension, high triglycerides, and low HDL-cholesterol.

OR, odds ratio; CI, confidence interval.
