# Supplementary figures and images for: Age- and Sex-Specific Relationships between Household Income, Education, and Diabetes Mellitus in Korean Adults: The Korea National Health and Nutrition Examination Survey, 2008-2010
Source: PLoS One. 2015 Jan 26;10(1):e0117034. doi: 10.1371/journal.pone.0117034 (PMC4306546; doi:10.1371/journal.pone.0117034)

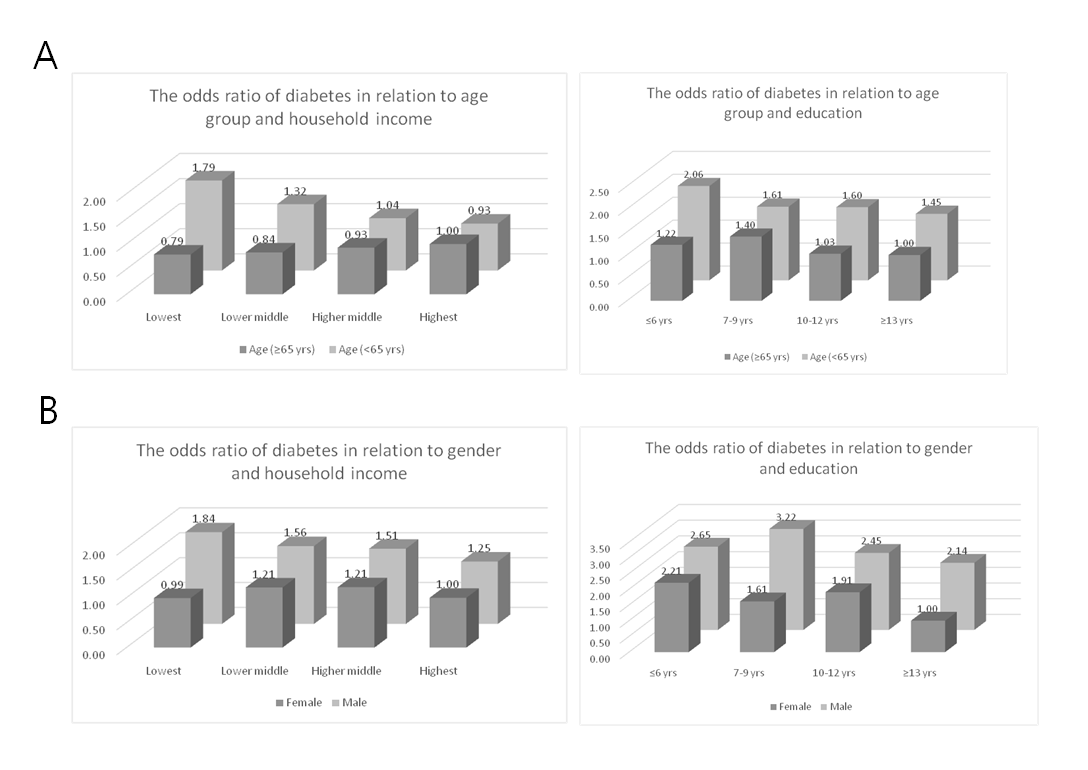

Supplement: S1 Fig — (TIF) [file pone.0117034.s003.tif]

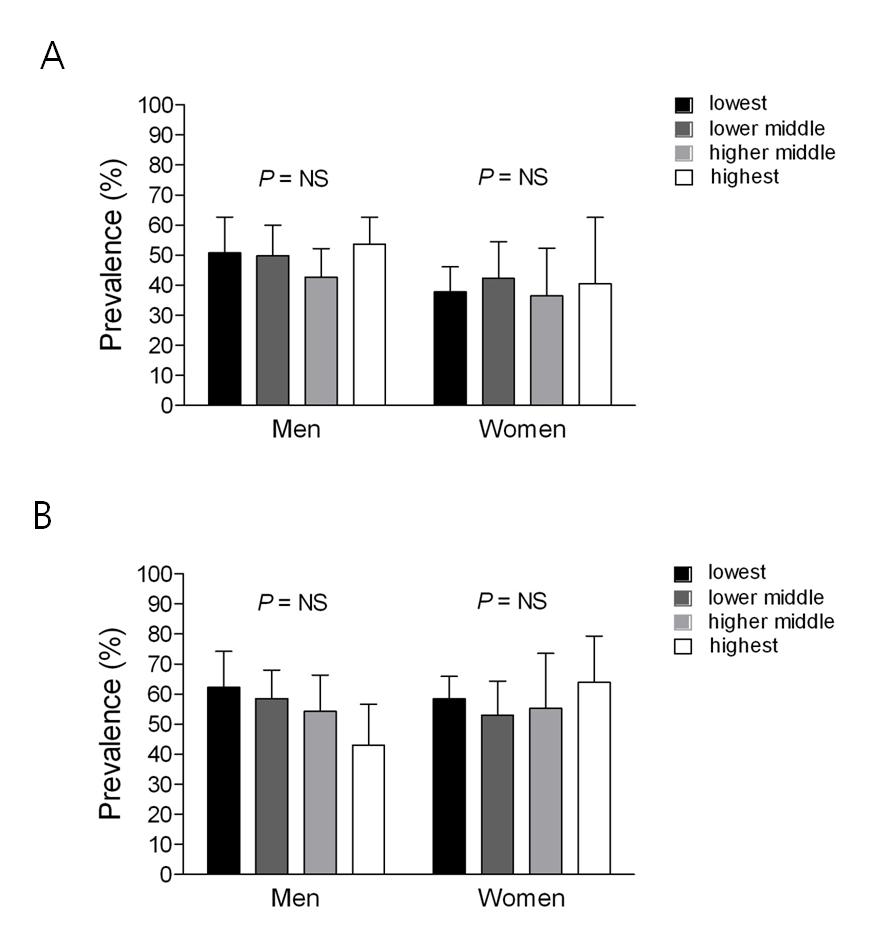

Supplement: S2 Fig — NS, non-specific. (TIF) [file pone.0117034.s004.tif]

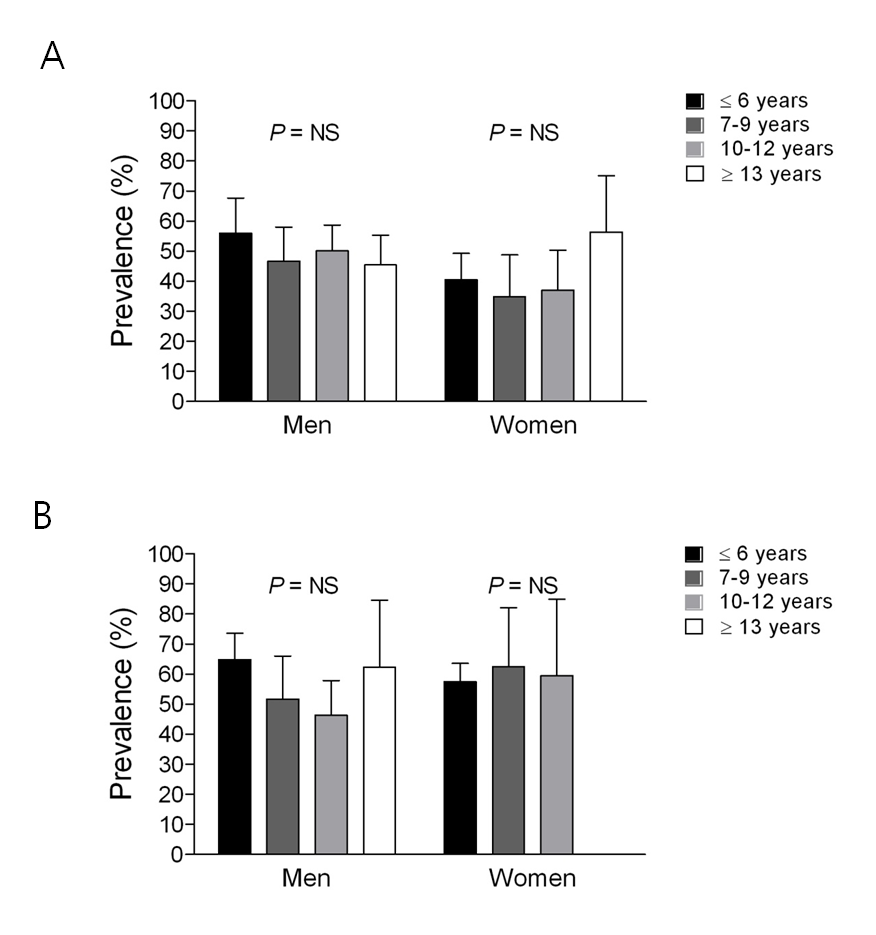

Supplement: S3 Fig — NS, non-specific. (TIF) [file pone.0117034.s005.tif]
